# The smallest angiosperm genomes may be the price for effective traps of bladderworts

**DOI:** 10.1093/aob/mcae107

**Published:** 2024-12-31

**Authors:** František Zedek, Jakub Šmerda, Aneta Halasová, Lubomír Adamec, Adam Veleba, Klára Plačková, Petr Bureš

**Affiliations:** Department of Botany and Zoology, Faculty of Science, Masaryk University, Kotlářská 2, 611 37 Brno, Czech Republic; Department of Botany and Zoology, Faculty of Science, Masaryk University, Kotlářská 2, 611 37 Brno, Czech Republic; Department of Botany and Zoology, Faculty of Science, Masaryk University, Kotlářská 2, 611 37 Brno, Czech Republic; Department of Experimental and Functional Morphology, Institute of Botany of the Czech Academy of Sciences, Dukelská 135, 37901, Třeboň, Czech Republic; Department of Botany and Zoology, Faculty of Science, Masaryk University, Kotlářská 2, 611 37 Brno, Czech Republic; Department of Botany and Zoology, Faculty of Science, Masaryk University, Kotlářská 2, 611 37 Brno, Czech Republic; Department of Botany and Zoology, Faculty of Science, Masaryk University, Kotlářská 2, 611 37 Brno, Czech Republic

**Keywords:** Carnivory, cytochrome oxidase, chromosome size, *Genlisea*, genome size, Lentibulariaceae, *Pinguicula*, recombination rate, smallest genomes, *Utricularia*

## Abstract

**Background:**

Species of the carnivorous family Lentibulariaceae exhibit the smallest genomes in flowering plants. We explored the hypothesis that their minute genomes result from the unique mitochondrial cytochrome *c* oxidase (COX) mutation. The mutation may boost mitochondrial efficiency, which is especially useful for suction-bladder traps of *Utricularia*, but also increase DNA-damaging reactive oxygen species, leading to genome shrinkage through deletion-biased DNA repair. We aimed to explore the impact of this mutation on genome size, providing insights into genetic mutation roles in plant genome evolution under environmental pressures.

**Methods:**

We compiled and measured genome and mean chromosome sizes for 127 and 67 species, respectively, representing all three genera (*Genlisea*, *Pinguicula* and *Utricularia*) of Lentibulariaceae. We also isolated and analysed COX sequences to detect the mutation. Through phylogenetic regressions and Ornstein–Uhlenbeck models of trait evolution, we assessed the impact of the COX mutation on the genome and chromosome sizes across the family.

**Results:**

Our findings reveal significant correlations between the COX mutation and smaller genome and chromosome sizes. Specifically, species carrying the ancestral COX sequence exhibited larger genomes and chromosomes than those with the novel mutation. This evidence supports the notion that the COX mutation contributes to genome downsizing, with statistical analyses confirming a directional evolution towards smaller genomes in species harbouring these mutations.

**Conclusions:**

Our study confirms that the COX mutation in Lentibulariaceae is associated with genome downsizing, probably driven by increased reactive oxygen species production and subsequent DNA damage requiring deletion-biased repair mechanisms. While boosting mitochondrial energy output, this genetic mutation compromises genome integrity and may potentially affect recombination rates, illustrating a complex trade-off between evolutionary advantages and disadvantages. Our results highlight the intricate processes by which genetic mutations and environmental pressures shape genome size evolution in carnivorous plants.

## INTRODUCTION

Genome size (the nuclear DNA content) in flowering plants varies over three orders of magnitude (Pellicer and Leitch, 2020). The proximate cause of this variation is polyploidy and the proliferation and removal of repetitive DNA, especially transposable elements (Leitch and Leitch, 2008; Tenaillon *et al.*, 2010; Lisch, 2013; Bennetzen and Wang, 2014; Šmarda *et al.*, 2019). Whether the size changes induced by polyploidy and/or transposable element dynamics become fixed depends on population dynamics and the effects of genome size change on fitness (Lynch and Conery, 2003; Knight *et al.*, 2005; Faizullah *et al.*, 2021; Bureš *et al.*, 2024). Genome size may, at least in part, determine the biology of the species, for example through its effects on cell size and the duration of cell division (Francis *et al.*, 2008; Šímová and Herben, 2012; Bhadra *et al.*, 2023). Conversely, the environmental pressure on cell size and the duration of cell division may select for genome size (Veselý *et al.*, 2012; Šmarda *et al.*, 2019). For example, the largest genomes are generally found in geophytes, where genome size growth via repetitive DNA appears to be selectively neutral (Veselý *et al.*, 2012; Šmarda *et al.*, 2019; Bureš *et al.*, 2024). On the opposite side of the spectrum, the smallest genome of 1C = 61 Mbp in flowering plants has thus far been found in *Genlisea tuberosa* (Fleischmann *et al.*, 2014) from the carnivorous family Lentibulariaceae, whose species generally exhibit small and minute genomes (Bennett and Leitch, 2005; Greilhuber *et al.*, 2006; Fleischmann *et al.*, 2014; Veleba *et al.*, 2014, 2020; Elliott *et al.*, 2022).

It has been hypothesized that nutrient-poor habitats, in which carnivorous plants generally occur (Givnish *et al.*, 1984, 2018), favour smaller genomes because of their lower demands on nitrogen and phosphorus (Hanson *et al.*, 2001; Veleba *et al.*, 2017). However, carnivorous plants as a whole do not appear to have smaller genomes than their non-carnivorous relatives (Veleba *et al.*, 2020). The miniature genomes are rather a specific feature of Lentibulariaceae (Veleba *et al.*, 2020). Sequencing of several *Genlisea* and *Utricularia* species has revealed very low amounts of repetitive DNA, fewer genes and shorter introns in their minute genomes (Ibarra-Laclette *et al.*, 2013; Leushkin *et al.*, 2013; Vu *et al.*, 2015; Silva *et al.*, 2019).

A potential mechanism behind the genome shrinkage in Lentibulariaceae may come from a unique mutation in the mitochondrial gene for cytochrome *c* oxidase (COX) (Jobson *et al.*, 2004; Laakkonen *et al.*, 2006; Albert *et al.*, 2010). This mutation is characterized by the presence of two contiguous cysteine residues (CC) in helix 3 of the COX subunit I and, thus far, has been found in all 21 analysed *Utricularia* species and in *Genlisea hispidula* (Jobson *et al.*, 2004). *Genlisea aurea* has cysteine and serine (CS) in this position and has probably lost the second cysteine (Jobson *et al*., 2004). *Genlisea violacea*, all five analysed *Pinguicula* species and all other flowering plants studied to date have an ancestral lysine and serine (LS) in this position (Jobson *et al.*, 2004). Although the CC mutation can increase the efficacy of traps by providing additional energy (Laakkonen *et al.*, 2006; Ellison and Gotelli, 2009), the presence of cysteine in both CC and CS, with its reactive thiol group, can simultaneously alter electron transport dynamics and redox balance, promoting the leakage of electrons and the generation of reactive oxygen species (ROS; Laakkonen *et al.*, 2006; Klomsiri *et al.*, 2011; Baba and Bhatnagar, 2018). ROS damage DNA by inducing point mutations and double-strand breaks (Albert *et al.*, 2010; Schubert and Vu, 2016), whose deletion-biased repairs would lead to genome downsizing (Albert *et al.*, 2010; Ibarra-Laclette *et al.*, 2013).

**Fig. 1.**
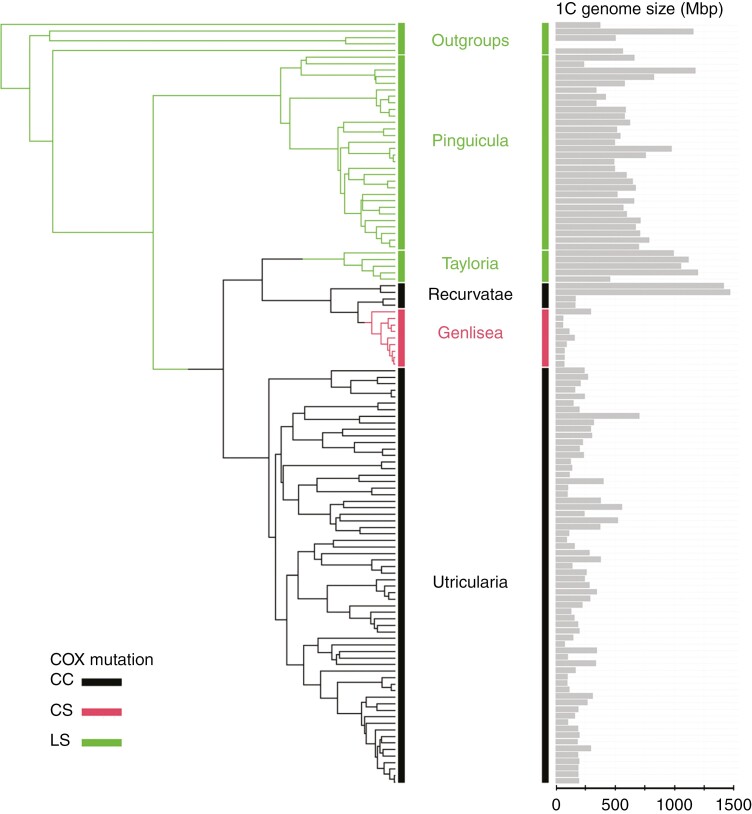
Ultrametric phylogenetic tree of analysed Lentibulariaceae species. In the case of the genus *Genlisea*, sections *Tayloria*, *Recurvatae* and *Genlisea* are depicted. The colours of the branches represent the state of the COX sequence, which is either the ancestral state of LS (green), CC mutation (black) or CS mutation (red). Grey bar plots show the genome size corresponding to the species of Lentibulariaceae and outgroups on the tips of the phylogenetic tree. Genome size for an outgroup species *Avicennia alba* (Acanthacae) is not available.

Although this hypothesis provided an elegant explanation for the minute genomes in Lentibulariaceae carrying the mutation, the test was performed on a very small and underrepresented dataset and did not find a link between small genomes in Lentibulariaceae species with the COX mutation (Veleba *et al.*, 2020). Here, we test the hypothesis on a representative species selection across all three Lentibulariaceae genera (*Genlisea*, *Pinguicula* and *Utricularia*; Fig. 1) by combining sequencing of the COX gene, genome size measurements complemented with database searches, and employing phylogenetically informed regressions and Ornstein–Uhlenbeck models of trait evolution.

## MATERIALS AND METHODS

### Plant material

Living plant species used in this study were provided from the private collection of Adam Veleba and the collection in the Institute of Botany CAS at Třeboň, Czech Republic, guided by Lubomír Adamec.

### Genome size measurements

We employed flow cytometry to measure the absolute genome size of *Genlisea roraimensis* N.E.Br. following the protocol of Veleba *et al.* (2014) using *Raphanus sativus* ‘Saxa’ with a genome size of 2C = 976 Mbp (Šmarda *et al.*, 2019) as a reference standard.

### Isolation of the COX sequences

Based on the sequences available in GenBank, we designed a pair of primers for PCR amplification of part of the coding region of COX subunit I containing the COX mutation site. The sequence of the forward primer was 5ʹ-GCCTGACATGGCATTTCCAC-3ʹ, and the sequence of the reverse was 5ʹ-GTTGTACGCACCTGGACCTTA-3ʹ. The PCR products were sequenced from both sides by Macrogen Inc. (The Netherlands). The raw sequences were manually processed, yielding a 237-bp (79 amino acids) long part of the coding region of COX subunit I that we inspected for the presence or absence of the COX mutation and aligned in MEGA X (Kumar *et al.*, 2018).

### Phylogenetic tree construction

To construct a phylogenetic tree of analysed Lentibulariaceae species, we used the OneTwoTree pipeline (Drori *et al.*, 2018) with default options and 100 bootstrap replicates. The list of markers that OneTwoTree used for the phylogenetic tree construction and their GenBank accession numbers are given in Supplementary Data Table S1. To obtain an ultrametric tree for subsequent phylogenetic analyses, the complete tree from OneTwoTree output (Table S2) was dated in MEGA X (Kumar *et al*., 2018) using the RelTime-Branch-lengths option (Tamura *et al.*, 2012; Mello, 2018) and setting *Avicenna alba*, *A. marina*, *Erythranthe guttata*, *Jacaranda mimosifolia* and *Verbena rigida* as outgroups. The obtained divergence times in the inferred ultrametric tree are relative as they were not calibrated. Finally, six species of *Genlisea* (*G. flexuosa*, *G. metallica*, *G. minor*, *G. nigrocaulis*, *G. oxycentron* and *G. tuberosa*) were added manually to the ultrametric tree based on published phylogenies and species affiliation to sections. This final ultrametric tree (Table S3) was pruned for the needs of each of the further analyses.

### Reconstruction of ancestral sequences

By sequencing and database searches, we obtained COX sequences from 73 Lentibulariaceae and five outgroup species (Supplementary Data Table S4). Of these 78 species, 55 were present on our ultrametric phylogenetic tree. To infer states of the COX mutation in internal nodes of the phylogenetic tree, we used the maximum-likelihood method of ancestral sequence reconstruction implemented in MEGA X (Kumar *et al.*, 2018). As an input, we used all the COX nucleotide sequences that we isolated or obtained from public databases (see above) and the phylogenetic tree we constructed using the OneTwoTree pipeline (see above). We employed the Jukes–Cantor nucleotide substitution model with a discrete Gamma distribution (JC+G) because this model was identified as the best substitution model based on the Bayesian information criterion (Table S5).

### Statistical analyses

To test whether species with or without the COX mutation differ in genome size or mean chromosome size, we employed phylogenetic regression as implemented in the R package Phylolm v.2.6.2 (Ho and Ane, 2014). We set the log_10_-transformed 1C genome size or mean chromosome size as a response variable and the state of the COX mutation (LS, CC and CS) as a categorical predictor. To select the best phylogenetic model for the regression’s error term, we used the weighted Akaike information criterion (AICw; Akaike, 1978; Wagenmakers and Farrel, 2004).

As a second approach in our investigation of genome size differences between species with and without the COX mutation, we utilized the R package OUwie v.2.10 (Beaulieu and OʹMeara, 2022). OUwie is a statistical tool used to examine if a trait (e.g. genome size and mean chromosome size) is being pulled towards an optimum value, represented by a central point called theta (θ). The strength of this pulling force is expressed by the alpha parameter (α), while random fluctuations around θ are captured by sigma squared (σ^2^). Our goal was to estimate θ as the optimum to which the genome size and mean chromosome size tend to evolve along the phylogeny of analysed species depending on the state of the COX gene. We considered seven different models in our analysis. Two of these models were Brownian motion models (BM1 and BMS), assuming a constant trait variance over time. The remaining five models were Ornstein–Uhlenbeck: OU1, OUM, OUMV, OUMA and OUMVA. Each of these models incorporates different levels of variation in the parameters θ, α and σ^2^. The selection of the best model was based on AICw. To evaluate the significance of the difference in the θ parameter between species with and without the COX mutation, we performed 100 parametric bootstrap replications using the ‘OUwie.boot’ function and tested the difference between the two θ distributions using the Mann–Whitney test. Finally, we used the R package ggplot2 (Wickham, 2016) to visualize distributions of bootstrapped genome size values in each category.

Due to the unavailability of materials for DNA isolation or sequences in public databases for all Lentibulariaceae species with known genome size, we inferred the COX sequence status for these species based on their phylogenetic affiliation within specific genera (*Pinguicula*, *Utricularia*) or sections (*Genlisea*). Although the available sequences indicated uniform COX mutations across entire genera (*Utricularia*, *Pinguicula*) or specific sections (*Genlisea*), the potential for bias exists in assigning mutations to species lacking COX sequence data. To mitigate this concern, we conducted all analyses twice: first, including species whose COX state was determined by both sequence data and phylogenetic affiliation, and second, restricting our analysis to only those species for which COX sequences were directly available.

## RESULTS

Altogether, we obtained genome size data for 18 *Genlisea*, 72 *Utricularia* and 37 *Pinguicula* species (Supplementary Data Table S6). These numbers represent 60, 30 and 32 % of the respective species diversity within these genera and encompass ~33 % of the species diversity in the family Lentibulariaceae (Angiosperm Phylogeny Group *et al.*, 2016). Of these 127 species, 112 with data on genome sizes and 67 with data on mean chromosome sizes were also on the constructed phylogenetic tree and, therefore, available for further analyses (Fig. 1; Table S6). Our phylogenetic tree (Fig. 1) was congruent with the phylogenies based on complete chloroplast (Li and Liu, 2022; Silva *et al.*, 2023) and mitochondrial genomes (Silva *et al.*, 2023).

The available COX sequences (Supplementary Data Table S6) and the ancestral sequence reconstruction (see Methods) showed that all analysed *Pinguicula* species exhibited the ancestral lysine–serine (LS) configuration at the site of interest (Fig. 1). The common ancestor of *Utricularia* and *Genlisea* underwent a mutation to cysteine–cysteine (CC), which is shared across all *Utricularia* species (Fig. 1). In *Genlisea*, only species from the subgenus *Genlisea* section *Recurvatae* retained the CC mutation (Fig. 1). The reversion to LS took place in the common ancestor of the subgenus *Tayloria*, and a mutation from CC to cysteine–serine (CS) occurred in the stem of the subgenus *Genlisea* section *Genlisea* (Fig. 1).

The phylogenetically corrected linear regression analysis revealed significant associations within Lentibulariaceae species. Species harbouring the ancestral LS mutation exhibited notably larger genomes (*P* << 0.001) and chromosomes (*P* << 0.001) compared to those with either CC or CS mutations (Table 1). Moreover, among species with mutations, those with the CS mutation displayed smaller chromosomes (*P* = 0.044) than those with the CC mutation. At the same time, no significant difference in genome size existed between these mutation categories (*P* = 0.144). Furthermore, the differences in the state of the COX sequence explained 16.52 and 28.63 % of the variance in the genome and mean chromosome size, respectively (Table 1). Notably, the best model for error terms in the phylogenetic regression was the Ornstein–Uhlenbeck model (OUfixedRoot; Supplementary Data Table S7A), suggesting a directionality in the evolution of the genome or mean chromosome size of Lentibulariaceae. The second best-fitting model for error terms in the phylogenetic regression was OUrandomRoot, with performance comparable to OUfixedRoot (Table S7A) and providing the same results as in the case of OUfixedRoot (compare Table 1 to Table S8A).

**Table 1.** Results of phylogenetic regressions of genome and mean chromosome size on the COX mutation under the OUfixedRoot model for the error term

Model terms	Estimate	s.e.	*t*-value	*P*-value	*R* ^2^ adjusted
**Regression model: log**_**10**_ **(1C genome size) ~ COX mutation**					
COX_LS (intercept)	2.794943	0.078011	35.8276	<<0.001	0.1652
COX_CC	−0.427816	0.094932	−4.5066	<<0.001	
COX_CS	−0.689525	0.193492	−3.5636	0.0005	
**Regression model: log**_**10**_ **(mean chromosome size) ~ COX mutation**					
COX_LS (intercept)	1.778338	0.092111	19.3065	<<0.001	0.2863
COX_CC	−0.560622	0.121249	−4.6237	<<0.001	
COX_CS	−0.951647	0.212225	−4.4841	<<0.001	

**Fig. 2.**
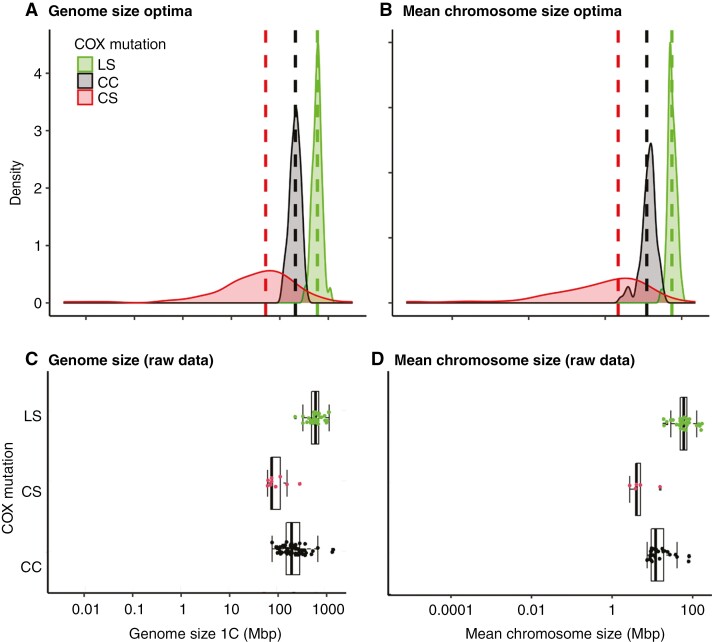
Densitograms of the bootstrapped values of (A) 1C genome size and (B) mean chromosome size optima in Lentibulariaceae species with the LS, CC and CS state of the COX sequence under the OUMA model. Vertical dashed lines indicate the estimated optima (Supplementary Data Table S10A). For comparison purposes, box plots of the raw genome size (C) and mean chromosome size data (D) overlaid by individual data points are also shown.

The presumed directionality in the genome or mean chromosome size evolution was confirmed in the OUwie analysis. The best-fitting model was an Ornstein–Uhlenbeck model, OUMA (Supplementary Data Table S9A), showing that the largest genome size (θ_LS_ = 597 Mbp) is linked to the ancestral state (LS) of the COX gene, while the CC and CS mutations are linked to significantly smaller genomes (*P* << 0.001; Mann–Whitney test), with θ_CC_ = 210 Mbp and θ_CS_ = 51.09 Mbp (Fig. 2A; Table S10A). OUMA was also the best-fitting model for mean chromosome size evolution (Table S9A). The largest mean chromosome size was estimated in species with the ancestral LS (θ_LS_ = 55.41 Mbp), while the CC and CS mutations are linked to significantly smaller chromosomes (*P* << 0.001; Mann–Whitney test), with θ_CC_ = 12.21 Mbp and θ_CS_ = 2.18 Mbp, respectively (Fig. 2B; Table S10A). The estimated genome and mean chromosome size optima (Fig. 2A, B) are close to the raw genome and mean chromosome size data for species with CC, CS and LS COX state (Fig. 2C, D).

When we performed phylogenetic regressions and OUwie analyses only with those species for which we had the COX sequences (while omitting those for which the COX state was estimated – see Methods and Supplementary Data Table S6), the results were congruent with the above analyses, showing the largest genomes and chromosomes in species with the ancestral LS and smaller genomes and chromosomes in species with CC and CS (Tables S8B and S10B).

## DISCUSSION

Our findings indicate that the whole genus *Utricularia* and sections *Recurvatae* and *Genlisea* from the genus *Genlisea* harbour CC or CS mutations in the COX gene (Fig. 1). Although these mutations may enhance the energetic power of mitochondria (Jobson *et al*., 2004; Laakkonen *et al.*, 2006; Ellison and Gotelli, 2009; Albert *et al.*, 2010), the presence of cysteine, with its reactive thiol group, can simultaneously alter electron transport dynamics and redox balance, promoting the leakage of electrons and the generation of ROS (Laakkonen *et al.*, 2006; Klomsiri *et al.*, 2011; Baba and Bhatnagar, 2018). The observation that these Lentibulariaceae lineages tend to evolve smaller genome sizes compared to those with the ancestral LS state (genus *Pinguicula*, *Genlisea* section *Tayloria*; Fig. 2) aligns with the hypothesis that changes in the COX sequence elevate ROS production, increasing DNA damage and fostering deletion-biased DNA repair, culminating in genome contraction (Albert *et al.*, 2010; Ibarra-Laclette *et al.*, 2013).

A more effective respiratory chain linked to the COX mutation goes hand in hand with the high energetic demands of many *Utricularia* species. Most extant *Utricularia* species possess sophisticated active traps (suction bladders), constantly pumping out water to maintain the negative pressure inside (Adamec, 2011; Vincent and Marmottant, 2011; Poppinga *et al.*, 2015). Many *Utricularia* species, the aquatic species being the typical example, exhibit rapid growth and remarkably high photosynthetic rates (Adamec, 2006, 2013). Small genomes would, therefore, be preferred in *Utricularia*, because, in plants, small genomes are associated with increased photosynthesis efficiency by allowing for smaller, more densely packed cells, which improves the leaf gas exchange capabilities (Roddy *et al.*, 2020). We can only speculate whether the COX mutation provided some advantage also to the common ancestor of *Utricularia* and *Genlisea* (Fig. 1), which had a rather passive trapping system (Westermeier *et al.*, 2017). Regardless of the trap’s form or function, the COX mutation was present in the ancestor (Fig. 1), altering the production of ROS, and thereby gradually reducing genome size anyway. Species with smaller genomes experience faster rates of cell division (Francis *et al.*, 2008; Šímová and Herben, 2012) and have lower phosphorus and/or nitrogen requirements (Šmarda *et al.*, 2013; Peng *et al.*, 2022), which could have been advantageous for rapidly growing *Utricularia* species regardless of the higher effectivity of their traps.

On the other hand, the extreme genome downsizing observed in *Utricularia* and *Genlisea* (Figs 1 and 2) might pose several disadvantages. Shrinkage of their genomes, probably driven by deletion-biased DNA double-strand break repair (Ibarra-Laclette *et al.*, 2013; Schubert and Vu, 2016), probably started with a reduction of non-essential DNA. Indeed, these miniature genomes have very few repetitive elements, indicating a drastic reduction in non-genic DNA (Ibarra-Laclette *et al.*, 2013; Vu *et al.*, 2015). However, further genome shrinkage threatens genes with the potential to reduce genetic variability, potentially limiting adaptability and evolutionary flexibility (Ibarra-Laclette *et al.*, 2013). Although whole-genome duplications can temporarily buffer against the loss of crucial genes, the repeated cycles of duplication followed by genome shrinkage might lead to a higher gene density, eventually increasing the likelihood of deleterious mutations anyway (Ibarra-Laclette *et al.*, 2013; Leushkin *et al.*, 2013). The balance between maintaining essential functions and enabling evolutionary innovation may thus be precarious in *Utricularia* and *Genlisea*.

**Fig. 3.**
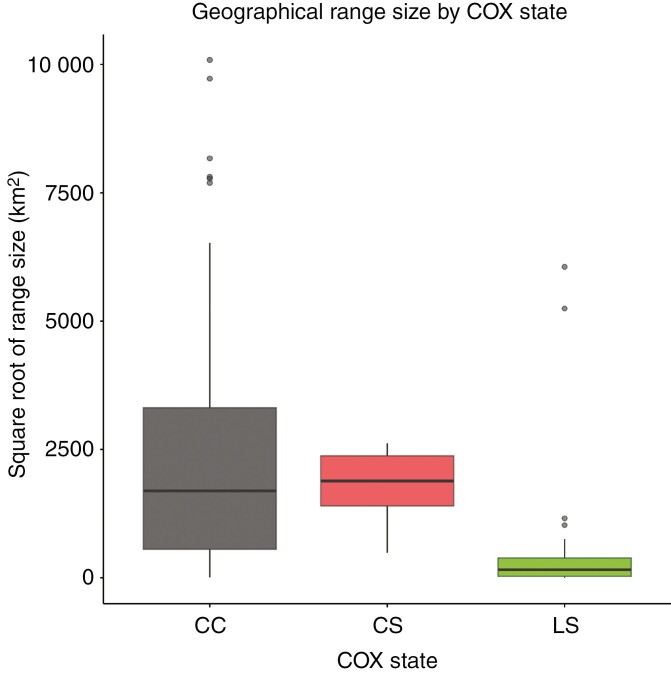
Differences in geographical range size between Lentibulariaceae species possessing either ancestral LS state of the COX gene, or CC and CS mutations. The data on range sizes were taken from Bureš *et al.* (2024).

There are at least three perspectives for future research. First, the amino acid changes in the COX protein specific to *Utricularia* and *Genlisea* (Fig. 1) necessitate in-depth functional studies. Second, the presumed correlation between ROS production, efficiency of double-strand break repair and genome size differences among Lentibulariaceae species requires direct measurement of DNA repair differences. If species with the COX mutation indeed experience increased ROS-induced double-strand breaks, it is also probable that they undergo higher recombination rates as a byproduct of DNA repair processes (Shrivastav *et al.*, 2008; Lieber, 2010). While recombination can promote adaptation across various environments, it can also disrupt advantageous allele combinations (Stapley *et al.*, 2017). Consequently, the impact of elevated recombination rates on fitness depends on the stability of the species’ environment (Ritz *et al.*, 2017). Finally, further research should focus on demography, speciation rates and other relevant measures of evolutionary success to elucidate whether the family Lentibulariaceae experiences more advantages or disadvantages from COX mutations. If the size of a species’ geographical range is considered a measure of success, then based on published data (Bureš *et al.*, 2024), species with COX mutations appear to fare better, as they tend to have larger ranges on average (Fig. 3). This is in accordance with the generally higher flexibility of species with smaller genomes and the disadvantages posed by large genomes (Knight *et al.*, 2005; Roddy *et al.*, 2020; Bhadra *et al.*, 2023; Bureš *et al.*, 2024).

## SUPPLEMENTARY DATA

Supplementary data are available at *Annals of Botany* online and consist of the following.

Table S1: Markers used for the phylogenetic tree construction with their respective GenBank accession numbers. Table S2: Phylogenetic tree of 120 Lentibulariaceae and outgroup species obtained from OneTwoTree in Nexus format. Table S3: Ultrametric phylogenetic tree (containing 126 Lentibulariaceae and outgroup species) that was used for analyses. Table S4: Accession numbers and database sources of the COX sequences from Lentibulariaceae and outgroups. Table S5: Maximum-likelihood fits of 24 different nucleotide substitution models for the COX sequences of Lentibulariaceae. Table S6: The main dataset used in all analyses. Table S7: Performance of phylogenetic models for the error term in the Phylolm analyses. Table S8: Results of phylogenetic regressions of the genome and mean chromosome size on the COX mutation with the second-best model for the error term (OUrandomRoot). Table S9: Performance of models of trait evolution in OUwie analyses based on AICw. Table S10: Parameters theta, alpha and sigma estimated under the OUMA model in the OUwie analyses.

mcae107_suppl_Supplementary_Material

## Data Availability

Data that were used to generate the results of the study are available in the supplementary data files online. Nucleotide COX sequences that were newly sequenced were deposited in GenBank under accession numbers OR987796–OR987833.
